# Complete Elimination of Peripheral Auditory Input Before Onset of Hearing Causes Long-Lasting Impaired Social Memory in Mice

**DOI:** 10.3389/fnins.2021.723658

**Published:** 2021-07-27

**Authors:** Rui Guo, Yang Li, Jiao Liu, Shusheng Gong, Ke Liu

**Affiliations:** Department of Otolaryngology Head and Neck Surgery, Beijing Friendship Hospital, Capital Medical University, Beijing, China

**Keywords:** hearing, hearing loss, congenital, learning memory, social memory, hippocampus

## Abstract

Hearing is one of the most important senses needed for survival, and its loss is an independent risk factor for dementia. Hearing loss (HL) can lead to communication difficulties, social isolation, and cognitive dysfunction. The hippocampus is a critical brain region being greatly involved in the formation of learning and memory and is critical not only for declarative memory but also for social memory. However, until today, whether HL can affect learning and memory is poorly understood. This study aimed to identify the relationship between HL and hippocampal-associated cognitive function. Mice with complete auditory input elimination before the onset of hearing were used as the animal model. They were first examined via auditory brainstem response (ABR) to confirm hearing elimination, and behavior estimations were applied to detect social memory capacity. We found significant impairment of social memory in mice with HL compared with the controls (*p* < 0.05); however, no significant differences were seen in the tests of novel object recognition, Morris water maze (MWM), and locomotion in the open field (*p* > 0.05). Therefore, our study firstly demonstrates that hearing input is required for the formation of social memory, and hearing stimuli play an important role in the development of normal cognitive ability.

## Introduction

Hearing is one of the most important senses needed for survival, and its loss is a highly prevalent sensory deficit in humans. Approximately 1.57 billion people in the world have hearing loss (HL), with increasing prevalence over the years (Haile et al., 2021). HL can lead to communication difficulties, social isolation, and cognitive dysfunction. A series of epidemiological studies have shown that HL may be an independent risk factor for dementia (Taljaard et al., 2016; Livingston et al., 2017; Loughrey et al., 2018; Griffiths et al., 2020). However, the underlying mechanism is still unclear.

The hippocampus serves as a critical brain region in the formation and maturation of learning and memory. A recent human study proposed that midlife HL can lead to atrophy of the hippocampus and entorhinal cortex (Armstrong et al., 2019), suggesting that HL may have negative effects on the hippocampus. Previous animal studies also showed that HL is able to disrupt the function of the hippocampus and lead to memory decline. The hippocampus is involved not only in declarative memory but also in social memory, which is the ability to remember and discriminate novel conspecifics from familiar ones in social activities (Barbara and Contreras, 1985). Although HL has been reported to negatively affect the quality of social interactions in patients (Hughes et al., 2018), these types of studies are usually limited because the patient samples are not quite uniform for precise analyses. Therefore, an animal model with diminished peripheral hearing input is necessary to verify the association between HL and social memory.

In this study, we created *Otof* mutation mice which are functionally equivalent to the otoferlin knockout mice (Otof^–/–^) via genetic manipulations. In this congenital deaf mouse model, the peripheral hearing input has been totally eliminated before hearing onset due to the complete loss of exocytosis in the cochlear ribbon synapses (Roux et al., 2006). Auditory brainstem response (ABR) examinations were used to confirm elimination of hearing, and the behavior estimations including three-chamber social interaction assay, novel object recognition, open field, and Morris water maze (MWM) were applied to detect the cognition and locomotion in this mouse model. We also found that mice with auditory input elimination before hearing onset have impaired social novelty memory, coupling with unimpaired locomotor activity and sociability. Thus, our study may firstly demonstrate that hearing input is required to establish social memory in mice.

## Materials and Methods

### Animals

Otof knockout mice were constructed by the CRISPR–Cas9 system. In brief, a frameshift mutation was introduced into the conserved domain (14 exon) of otoferlin using the AAV loading Cas9 protein and sgRNA (GTGAAAATTTACCGAGCAGA), respectively. The mutation site was identified by Sanger sequencing. The heterozygous animals were interbred to generate Otof^+/+\u0001^, Otof^±^, and Otof^–/–^ mice. C57BL/6J mice served as the control group and were purchased from the Vital River Laboratory (Beijing, China). All mice were bred in the Experimental Animal Department of the Capital Medical University at 22–25°C, 50% humidity, and 12 h light–dark cycle, with food and water available *ad libitum*. Only male mice were used in this study. The animal study was reviewed and approved by the Animal Ethics Committee of the Capital Medical University.

### Assessment of Auditory Function

Auditory brainstem response detection was performed at the ages of P14, P56, and P168. Mice were anesthetized with an intraperitoneal injection of ketamine (100 mg/kg, Sigma, Saint Louis, MO, United States) plus xylazine (10 mg/kg, Sigma), and then placed in an electrically shielded and soundproofed audiometric chamber (Shanghai Shengnuo Acoustic Equipment, Shanghai, China). Meanwhile, body temperature was maintained with a constant temperature heating pad. The needle electrodes were placed subcutaneously, with a reference electrode beneath the pinna of the tested ear, a recording electrode (+) at the junction of anterior edges of both auricles and the midline of the cranial apex, and a ground electrode in the contralateral ear. Acoustic stimuli were delivered monaurally by an earphone attached to a customized plastic speculum inserted into the ear canal. Calibrated tone bursts with 5 ms duration and 0.5 ms rise–fall time were synthesized and presented using TDT System 3 hardware and SigGen/BioSig software (Tucker-Davis Technologies, Alachua, FL, United States). ABRs were measured at click and tong burst. A total of 1,024 responses were averaged near the threshold at various intensities with 5 dB intervals. The lowest level at which ABR waves could be clearly detected was defined as the threshold.

### Immunostaining and Confocal Microscopy

Cochleas of the mice were perfused with 4% paraformaldehyde (PFA) in PBS (pH 7.4) and incubated in the same fixative at 4°C, overnight. The cochleas were rinsed three times with PBS and decalcified by incubation with 10% ethylenediamine tetraacetic acid (EDTA) for 4–6 h. The organ of Corti was dissected into a surface preparation, preincubated in 0.3% Triton X-100 and 5% normal goat serum in PBS at room temperature for 1 h, and incubated overnight at 4°C, with the primary antibody:mouse anti-otoferlin antibody (1:300, Abcam, Cambridge, MA, United States, ab53233). The samples were rinsed three times in PBS buffer and incubated at room temperature for 2 h with the appropriate secondary antibody:goat anti-mouse Alexa Fluor 488. The samples were washed three times in PBS and mounted on a glass slide using a fluorescent mounting medium with DAPI (ZSGB-BIO, ZLI-9557). Fluorescence confocal z stacks of the organ of Corti were obtained with a Leica scanning laser confocal microscope (model TCS SP8 II, Leica, Wetzlar, Germany), equipped with a high-resolution objective (numerical aperture of 1.18, × 63 oil-immersion objective). Images were acquired in a 1,024 × 512 (pixel size = 0.036 μm in *x* and *y*) from top to bottom with an interval of 0.5 μm/layer.

For otoferlin staining on hippocampal slices, after anesthesia and cardiac perfusion with precooling PBS, the mouse brain was quickly removed and fixed in 4% PFA at 4°C, for 24 h, and then transferred into 30% sucrose for cryoprotection. After that, the brain was subjected to OCT embedding (Tissue-Tek, Torrance, CA, United States, 4853) and sliced into 40 μm coronal sections by a cryostat microtome (Leica CM3050S, Wetzlar, Germany) at −20°C,. The floating brain sections were washed in PBS three times for 10 min and blocked in 10% goat serum diluted in 0.3% Triton X-100 for 1 h at room temperature. After the block, the brain sections were incubated with primary antibody overnight at 4°C, and then incubated with secondary antibody for 2 h at room temperature. After three times washing with PBS, the sections were mounted on the glass slides with mounting medium with DAPI. Primary antibodies used in this study were rabbit anti-MAP2 (1:500, Abcam, ab32454) and mouse anti-otoferlin (1:100, Abcam, ab53233). Secondary antibodies used were goat anti-mouse Alexa Fluor 488 (1:500, Invitrogen, Waltham, MA, United States, A11029) and goat anti-rabbit Alexa Fluor 594 (1:500, Invitrogen, A11037). Images were taken by a confocal microscope (Leica TCS SP8).

### Three-Chamber Social Interaction Experiment

The three-chamber social interaction test was adapted from previous research (Wang et al., 2018). The three-chamber apparatus (Xinruan, Shanghai, China) was divided into three compartments (each compartment *L* × *W* × *H*: 20 cm × 40 cm × 22 cm). Mice can freely explore the three chambers through two doors (W × H: 5 cm × 8 cm). Briefly, the social interaction experiment included three continuous phases with two small wire cages (15 cm height with a diameter of 7 cm) in the left and right chamber. In the first phase, two empty wire cages were placed in the left and right chamber, respectively, and the WT or Otof^–/–^ mouse was introduced into the middle chamber and allowed to explore the apparatus freely for 10 min. In the second phase (social interaction test), an age-matched C57BL/6J male mouse (stranger 1, S1) was restricted into one of the wire cages randomly. The WT or Otof^–/–^ mouse was allowed to explore the apparatus freely for 10 min. After the second 10 min, in the third phase (social novelty test), another age-matched C57BL/6J male mouse (stranger 2, S2) was restricted into the previously empty wire cage, and the subject mouse was allowed to explore for 10 min. The close interaction area was defined as the surrounding area (20 cm × 17.5 cm) containing the wire cage. Time spent in each chamber by the subject mouse was recorded by SuperMaze software (Xinruan).

### Morris Water Maze

The MWM test was adapted from previous research (Hunt et al., 2013). The core device for MWM is a circular pool (120 cm in diameter) filled with non-toxic paint mixed with water. A circular platform (10 cm in diameter) was located in one quadrant of the pool above or beneath the water surface. Briefly, mice were trained to find and land a visible platform on day 1, and the escape latency (first time to land the platform) and swimming speed were evaluated to exclude those mice with visual impairment or dyskinesia. Then, the platform was fixed in another quadrant and beneath the water surface 0.5–1 cm. Mice were trained to learn and remember the location of the platform according to spatial cues around the pool for five sessions (days 2–4). Two daily sessions were ∼3.5 h interval. Each session included four trials, and mice were introduced into the water from four different start points and were allowed to swim for 60 s. Once the mouse landed the platform or did not find the platform within 60 s, it was allowed to stay on the platform for 30 s to remember the platform position. The probe was performed 24 h after the last training in session 5. The platform was removed and the mice were allowed to swim in the pool for 60 s. The percent time (%) in the platform quadrant and the first time to the platform by subject mice were recorded and used in the statistical analysis. The software used in this test was Smart v3.0 (Panlab Harvard Apparatus, Barcelona, Spain).

### Novel Object Recognition

Novel object recognition (Wang et al., 2020) was performed in the open arena (*L* × *W* × *H*: 50 cm × 50 cm × 50 cm). The task included two phases: acquisition phase and retrieval phase. In the acquisition phase, two similar objects (object A and A′) were placed in two corners of the arena; the mouse was gently placed into the open field and was allowed to explore for 10 min, and then the mouse came back to its home cage. Four hours later, in the retrieval phase, one of the objects (A) was replaced by a novel object (object B). The mouse was put back to the arena for another 10 min. The exploration time of each object in the retrieval phase was recorded by the SuperMaze software (Xinruan). The discrimination index (DI) was time with (object B - object A)/(object B + object A).

### Open Field

The open field test was performed as described previously (Wang et al., 2020) to test the spontaneous motor activity. The mouse was placed in the open field (*L* × *W* × *H*: 50 cm × 50 cm × 50 cm) for 5 min. The speed and distance of the test mouse was recorded by the SuperMaze software (Xinruan).

### Statistical Analysis

Statistical analysis was performed by GraphPad Prism 8.0.1 software. For two groups, data were analyzed by unpaired *t*-test. One-/two-way ANOVA was used for the comparison of more than two groups.

## Results

### Auditory Detection Confirms Complete Elimination of Hearing Input in Mice

**FIGURE 1:**
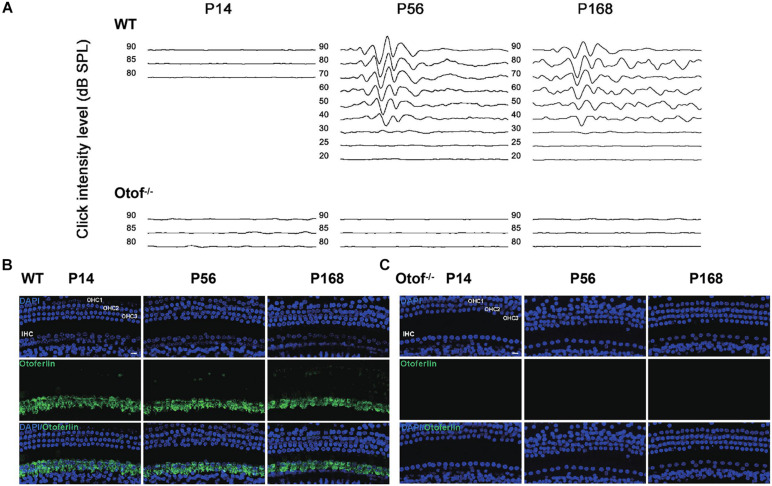
Complete elimination of hearing input presented before and after hearing onset in Otof^− /−^ mice. **(A)** No visible ABR waveforms (click) were found at P14, P56, and P168 in Otof^− /−^ mice, respectively; also, no ABR waveforms (click) were seen at P14 in WT mice. However, there are normal ABR waveforms (click) at P56 and P168 in WT mice. **(B)** Normal otoferlin expressions were seen in cochlear inner hair cells (IHCs) at P14, P56, and P168 in WT mice (green). **(C)** No positive immunostaining signals were found at P14, P56, and P168 in Otof^− /−^ mice. Bar = 10 μm; WT, wild-type mice; Otof^− /−^, otoferlin knockout mice; dB, decibel.

To confirm the complete elimination of hearing function in Otof^–/–^ mice in this study, we then detected the ABR thresholds at ages of P14, 56, and 168, respectively. No visible ABR waveforms were detected at any point (Otof^–/–^, Figure 1A), and normal waveforms were detected in the control mice (WT, Figure 1A). This shows that peripheral hearing input was eliminated, and no hearing information was being delivered from the ear to the brain in these deaf mice. In addition, we estimated the ABR threshold across frequencies in both Otof^–/–^ and WT mice. No ABR thresholds could be identified in Otof^–/–^ mice across all frequencies, whereas normal thresholds were observed in the control ones (Table 1). Otof^–/–^ mice can serve as complete deaf models without peripheral hearing input, similar to those previously reported (Roux et al., 2006). Furthermore, we detected otoferlin expression in the cochlear hair cells of Otof^–/–^ and WT mice at P14, P56, and P168. There were no positive immunostaining signals in Otof^–/–^ mice (Figure 1C), whereas normal expression was observed in the control ones (Figure 1B), confirming complete hearing elimination.

**TABLE 1:** Click and tone burst examinations in both WT and Otof^–/–^ mice.

Intensity level (dB SPL)	Click	4 kHz	8 kHz	16 kHz	24 kHz
**WT**					
90	Y	Y	Y	Y	Y
80	Y	Y	Y	Y	Y
70	Y	Y	Y	Y	Y
60	Y	Y	Y	Y	Y
50	Y	Y	Y	Y	Y
40	Y	Y	Y	Y	Y
30	Y	Y	Y	Y	Y
25	**Y**	**Y**	Y	Y	Y
20	N	N	Y	Y	Y
15			Y	Y	Y
10			**Y**	**Y**	**Y**
**Otof^–/–^**					
90	N	N	N	N	N
85	N	N	N	N	N
80	N	N	N	N	N

*In WT mice, the thresholds of click and 4, 8, 16, and 32 kHz are 25, 25, 10, 10, and 10 dB SPL, respectively. In Otof^–/–^ mice, no detectable thresholds are found at click, 4, 8, 16, and 32 kHz. Y means “Yes”; N means “No.”*

### Elimination of Hearing Input Causes Impaired Social Memory

**FIGURE 2:**
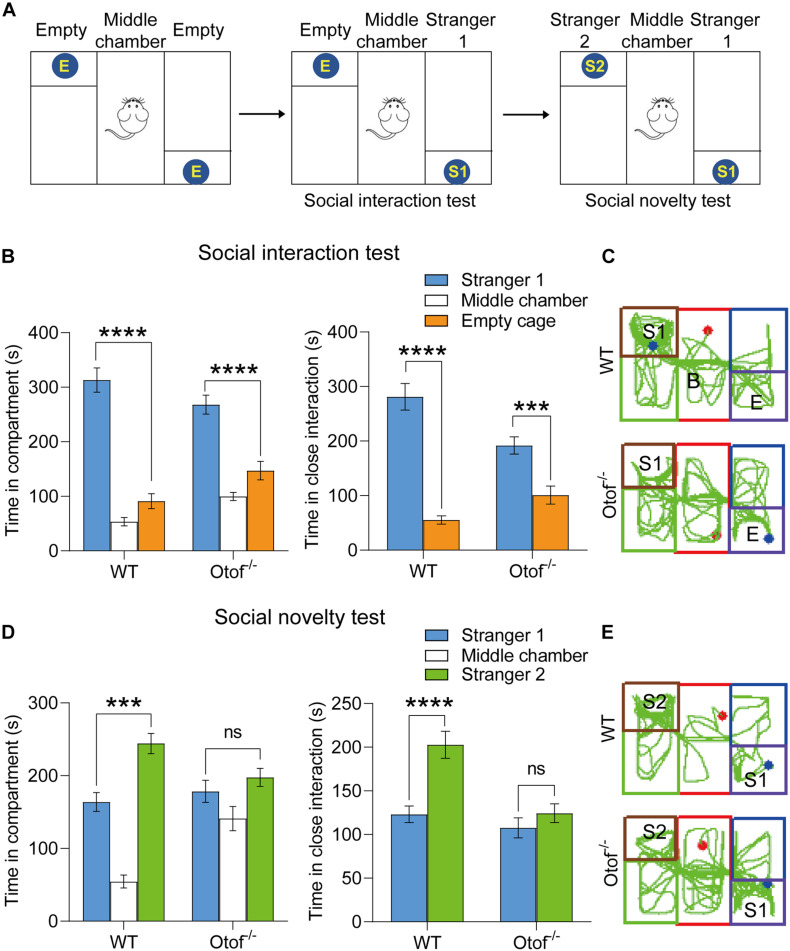
Otof^− /−^ mice showed impaired social memory in a three-chamber social interaction experiment at 2 months old. **(A)** The schematic diagram of a three-chamber social interaction experiment. **(B)** Both WT and Otof^− /−^ mice showed normal sociability in the social interaction test: the subject mouse spent more time interacting with stranger 1 (S1) than the empty wire cage E. **(C)** Representative tracks of WT and Otof^− /−^ mice in the social interaction test. **(D)** Otof^− /−^ mice showed impaired social memory in the social novelty test compared with WT mice. **(E)** Representative tracks of WT and Otof^− /−^ mice in the social novelty test. The starting point and the ending point of tracks in **(C,E)** were represented by a red dot and a green dot, respectively. All values are presented as mean ± SEM and analyzed by unpaired *t*-test for **(B)** and **(D)**. ***p < 0.001, ****p < 0.0001 vs. WT. WT: *n* = 20; Otof^− /−^: *n* = 21. WT, wild-type mice; Otof^− /−^, otoferlin knockout mice; E, empty; S1, stranger 1; S2, stranger 2; ns, not significant.

To explore whether HL can alter the hippocampal-dependent social interaction, a three-chamber social interaction experiment was used to sequentially test sociability and social novelty (Figure 2A). In this test, 2-month-old mice were allowed to explore either a chamber containing a stranger mouse (stranger 1, S1) restrained in a wire cage or the other chamber with an empty wire cage for 10 min. Both WT and deaf mice spent more time interacting with the S1 than the empty cage according to bilateral chambers (WT: 313.189 ± 22.303 vs. 91.147 ± 13.700 s, *n* = 20, ^****^p < 0.0001, *t*-test; Otof^–/–^: 268.093 ± 17.306 vs. 147.071 ± 16.959 s, *n* = 21, ^****^p < 0.0001, *t*-test; Figures 2B, C) and close interaction measurements (WT: 281.170 ± 24.467 vs. 55.398 ± 7.569 s, *n* = 20, ^****^p < 0.0001, *t*-test; Otof^–/–^: 191.996 ± 15.949 vs. 100.990 ± 16.590 s, *n* = 21, ^∗∗∗^*p* < 0.001, *t*-test; Figures 2B, C), indicating that deaf mice also have similar sociability as WT mice. Immediately after the social interaction test, we placed another novel mouse (stranger 2, S2) into the previously empty cage. The subject mouse was allowed to explore the three chambers for another 10 min. Taking advantage of the natural novelty instincts in rodents, both in bilateral chambers and close interaction measurements, WT mice spent more time exploring the S2 than S1 (time in compartment: 244.259 ± 13.968 vs. 163.984 ± 13.008 s, ^∗∗∗^*p* < 0.001, *t*-test; time in close interaction: 202.713 ± 15.540 vs. 123.043 ± 9.475 s, ^****^p < 0.0001, *t*-test; *n* = 20, Figures 2D, E). However, deaf mice spent comparable time between S1 and S2 without significant differences (time in compartment: 197.645 ± 12.500 vs. 178.459 ± 15.198 s, *p* > 0.05, *t*-test; time in close interaction: 124.299 ± 10.728 vs. 107.609 ± 11.491 s, *p* > 0.05, *t*-test; *n* = 21, Figures 2D, E). Thus, our study indicated that the social memory in deaf mice was significantly impaired.

### Elimination of Hearing Input Causes Long-Lasting Impaired Social Memory

**FIGURE 3:**
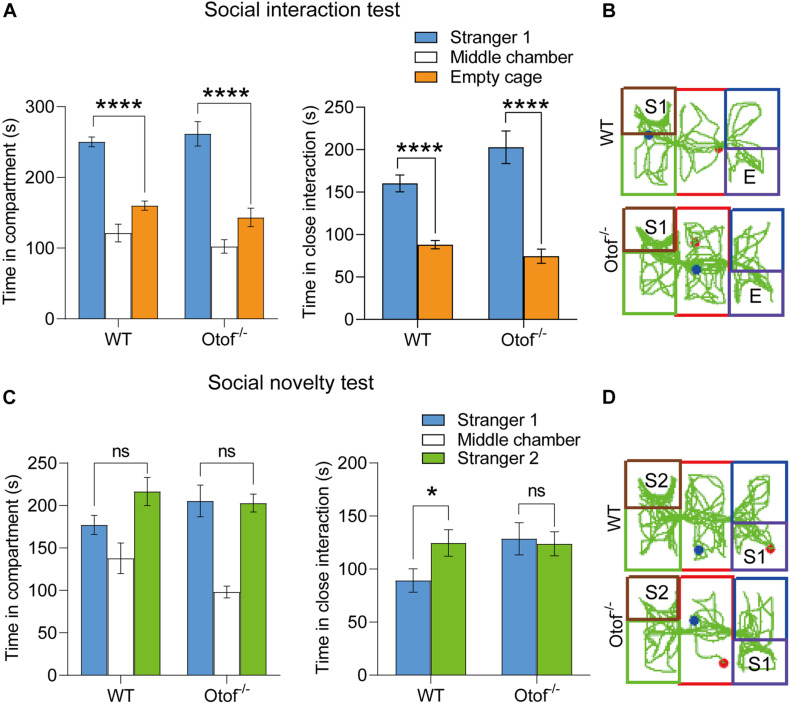
Otof^− /−^ mice showed impaired social memory in a three-chamber social interaction experiment at 6 months old. **(A)** WT and Otof^− /−^ mice showed normal sociability in the social interaction test. **(B)** Representative tracks of WT and Otof^− /−^ mice in the social interaction test. **(C)** Otof^− /−^ mice showed impaired social memory in the social novelty test. **(D)** Representative tracks of WT and Otof^− /−^ mice in the social novelty test. The starting point and the ending point of tracks in **(B,D)** were represented by a red dot and a green dot, respectively. All values are presented as mean ± SEM and analyzed by unpaired *t*-test for panels **(A,C)**. *p < 0.05, ****p < 0.0001 vs. WT. WT: *n* = 10; Otof^− /−^: *n* = 10. WT, wild-type mice; Otof^− /−^, otoferlin knockout mice; E, empty; S1, stranger 1; S2, stranger 2; ns, not significant.

Although we have found the impaired social memory in this study, however, we still do not know whether the impairment of social memory is transient or long-lasting. We then performed a three-chamber test in both deaf and control mice at 6 months of age. Consistent with the previous results at 2 months, the deaf mice showed normal sociability in bilateral chambers (WT: 250.302 ± 6.883 vs. 160.178 ± 6.490 s, *n* = 10, ^****^p < 0.0001, *t*-test; Otof^–/–^: 261.633 ± 17.249 vs. 143.451 ± 13.093 s, *n* = 10, ^****^p < 0.0001, *t*-test; Figures 3A, B) and close interaction (WT: 160.269 ± 9.927 vs. 88.121 ± 4.935 s, *n* = 10, ^****^p < 0.0001, *t*-test; Otof^–/–^: 202.845 ± 19.163 vs. 74.424 ± 8.319 s, *n* = 10, ^****^p < 0.0001, *t*-test; Figures 3A, B). In the social novelty test, although WT mice were unable to display a time preference in bilateral chambers (time in compartment: 216.517 ± 16.661 vs. 177.134 ± 11.296 s, *n* = 10, *p* > 0.05, *t*-test; Figures 3C, D), they had a distinct preference for close social interaction (time in close interaction: 124.593 ± 12.547 vs. 89.195 ± 11.004 s, *n* = 10, ^∗^*p* < 0.05, *t*-test; Figures 3C, D). However, in this study, the deaf mice (age: 6 months) were still unable to distinguish the S2 from S1 in bilateral chambers (time in compartment: 202.872 ± 10.463 vs. 205.414 ± 18.567 s, *n* = 10, *p* > 0.05, *t*-test; Figures 3C, D) and close interaction (time in close interaction: 123.763 ± 11.396 vs. 128.570 ± 15.194 s, *n* = 10, *p* > 0.05, *t*-test; Figures 3C,D), suggesting a long-lasting impaired social memory in deaf mice.

### Elimination of Hearing Input Is Unable to Affect Novel Object Recognition

**FIGURE 4:**
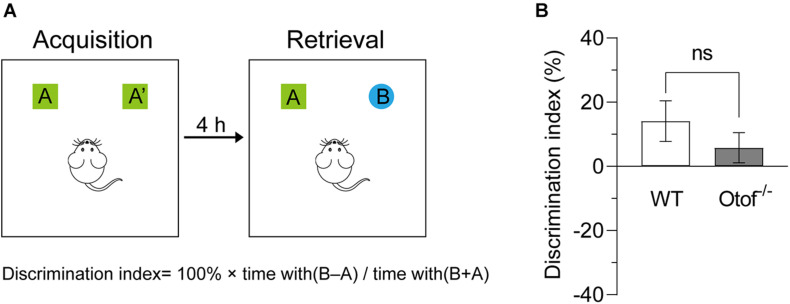
Otof^− /−^ mice showed unimpaired novel object recognition. **(A)** Experiment diagram of the novel object recognition test. **(B)** Compared with WT mice, Otof^− /−^ mice showed a comparable object discrimination index (DI). All values are presented as mean ± SEM and analyzed by unpaired *t*-test. WT: *n* = 11; Otof^− /−^: *n* = 14. WT, wild-type mice; Otof^− /−^, otoferlin knockout mice; ns, not significant.

To examine whether the HL affects the ability of object novelty, we carried out a novel object recognition test in both deaf and control mice at 2 months of age (Figure 4A). In this study, the DI showed that there is no significant difference between the groups. Deaf mice exhibited normal hippocampal-dependent object recognition capacity (5.771 ± 4.702 vs. 14.09 ± 6.346%, *p* > 0.05, *t*-test, WT: *n* = 10, Otof^–/–^: *n* = 14; Figure 4B).

### Elimination of Hearing Input Does Not Disrupt Spatial Learning and Memory

**FIGURE 5:**
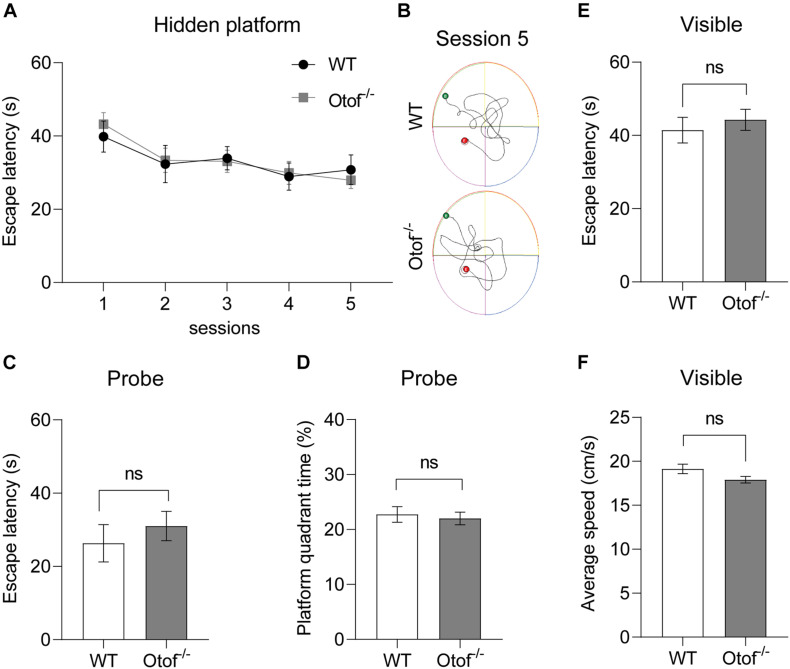
Otof^− /−^ mice showed normal spatial learning and memory in the Morris water maze (MWM). **(A)** The escape latency (time to find the hidden platform) recorded in training sessions. **(B)** Representative swimming trace of WT and Otof^− /−^ mice in session 5. **(C)** The escape latency (s) in the probe. **(D)** The percent time (%) in the platform quadrant in the probe. **(E)** The escape latency (s) in the visible platform period. **(F)** The average swimming speed (cm/s) in the visible platform period. All values are presented as mean ± SEM and analyzed by unpaired *t*-test for **(C–F)** and two-way ANOVA for panel **(A)** vs. WT. WT: *n* = 12; Otof^− /−^: *n* = 17. WT, wild-type mice; Otof^− /−^, otoferlin knockout mice; ns, not significant.

To explore whether the HL affects the capacity of spatial learning and memory, here, we use classic assessment of the MWM to estimate hippocampal-dependent spatial learning and memory in both deaf and control mice at 2 months of age. No significant differences in the visible platform test of escape latency (44.27 ± 2.868 vs. 41.42 ± 3.518 s, *p* > 0.05, *t*-test, WT: *n* = 12, Otof^–/–^: *n* = 17; Figure 5E) and swimming speed (17.91 ± 0.3840 vs. 19.13 ± 0.5327 cm/s, *p* > 0.05, *t*-test, WT: *n* = 12, Otof^–/–^: *n* = 17; Figure 5F) were found between both groups. Furthermore, no differences were found in escape latency in hidden platform training (Figures 5A,B). In the probe test after withdrawing platform, no significant differences were identified in the first-time arriving platform (31.03 ± 3.989 vs. 26.32 ± 5.092 s, *p* > 0.05, *t*-test, WT: *n* = 12, Otof^–/–^: *n* = 17; Figure 5C) and the percentage of platform quadrant (22.01 ± 1.150 vs. 22.74 ± 1.409%, *p* > 0.05, *t*-test, WT: *n* = 12, Otof^–/–^: *n* = 17; Figure 5D) between the two groups.

### Elimination of Hearing Input Is Unable to Disrupt Locomotion in the Open Field

**FIGURE 6:**
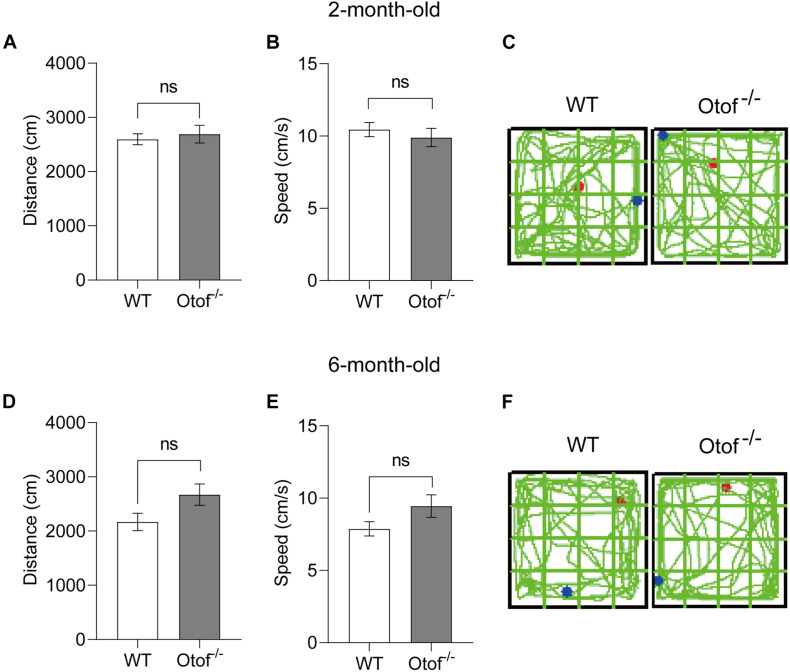
Otof^− /−^ mice showed normal motor ability in the open field (*L* × *W*: 50 × 50 cm) at the ages of 2 and 6 months. **(A,D)** In the open field, there was no significant difference in the exploration distance (cm) between WT and Otof^− /−^ mice group. **(B,E)** WT and Otof^− /−^ mice showed no significant difference of speed (cm/s). **(C,F)** Representative exploration traces of WT mice and Otof^− /−^ mice. The starting point and ending point of tracks in panels **(C, F)** were represented by a red dot and a green dot, respectively. All values are presented as mean ± SEM and analyzed by unpaired *t*-test for panels **(A,B,D,E)**. 2-month-old: WT, *n* = 15; Otof^− /−^, *n* = 17. 6-month-old: WT, *n* = 10; Otof^− /−^, *n* = 10. WT, wild-type mice; Otof^− /−^, otoferlin knockout mice; ns, not significant.

To exclude the possibility of motor alteration between the deaf and control mice groups, in this study, the open field testing was carried out to detect the locomotion distance (cm) and speed (cm/s) during a 5-min free exploration (Figure 6). No significant differences were found in the distance between the two groups, irrespective of age (2 months: 2,692 ± 163.2 vs. 2,597 ± 103.0 cm, *p* > 0.05, *t*-test, WT: *n* = 15, Otof^–/–^: *n* = 17; 6 months: 2,672 ± 197.1 vs. 2,169 ± 159.8 cm, *p* > 0.05, *t*-test, WT: *n* = 10, Otof^–/–^: *n* = 10; Figures 6A, D). Similarly, there was no significant difference in the mean speed (2 months: 9.897 ± 0.6342 vs. 10.44 ± 0.4822 cm/s, *p* > 0.05, *t*-test, WT: *n* = 15, Otof^–/–^: *n* = 17; 6 months: 9.446 ± 0.7823 vs. 7.871 ± 0.4931 cm/s, *p* > 0.05, *t*-test, WT: *n* = 10, Otof^–/–^: *n* = 10; Figures 6B, E), suggesting that HL is unable to affect spontaneous locomotor activity.

### Otoferlin Expression Is Nearly Undetectable in the Hippocampus of WT Mouse Brain

**FIGURE 7:**
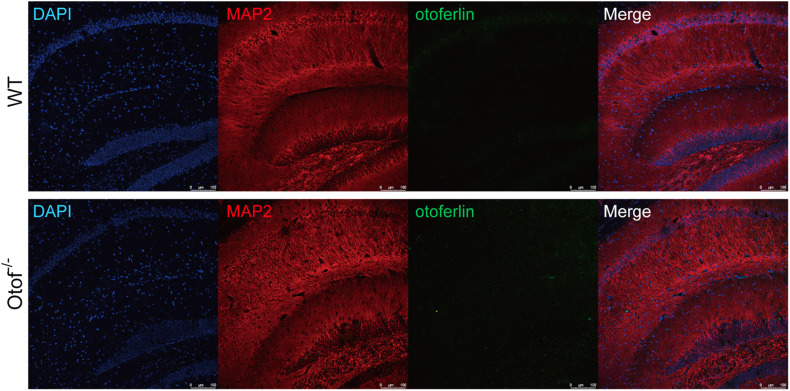
Otoferlin staining in the hippocampus of WT and Otof^− /−^ mice. Immunofluorescence of WT and Otof^− /−^ hippocampus slices with otoferlin (green), MAP2 (red), and DAPI (blue). WT, wild-type mice; Otof^− /−^, otoferlin knockout mice. Scale bars: 100 μm.

It has been once reported that otoferlin could be expressed in the brain (Wu et al., 2015). In order to exclude the possible effects on the cognitive function that otoferlin may have, we then investigated the expression level of otoferlin in the hippocampus. We performed otoferlin staining on hippocampal slices, and we did not identify clear positive signals of otoferlin expression in both WT and Otof^–/–^ mice (Figure 7), suggesting an excluded effect of hippocampal otoferlin expression on social memory.

## Discussion

In this study, we found that mice with auditory input elimination before hearing onset have impaired social memory and unimpaired sociability, novel object recognition, spatial learning, memory, and locomotor activity. This result shows that hearing input might be necessary to establish social memory in mice.

A significant number of studies have proposed that HL can lead to cognitive impairment, especially in the aspects of learning and memory (Beckmann et al., 2020; Griffiths et al., 2020; Slade et al., 2020). Under the impoverished auditory environments, HL patients have fewer chances to access normal social interactions. In addition, poor long-term social interactions are also risk factors for dementia (Kuiper et al., 2015). However, an investigation has been carried out to explore the relationships between HL and social memory; particularly, until today, no study has investigated the potential links between them using animal models. Therefore, to the best of our knowledge, this is the first study that focused on the relationship between the two via a congenital deaf mouse model, in which the peripheral hearing input was eliminated before its onset.

It has been fully reported that otoferlin is a critical synaptic protein, which specifically expresses in the inner hair cells (IHCs) of a mature cochlea (Roux et al., 2006). It interacts with SNARE to form a functional complex to account for neurotransmitter exocytosis at the ribbon synapses (Roux et al., 2006). Otof knockout or equivalent genetic manipulations can completely abolish neurotransmitter exocytosis, leading to profound HL (Yasunaga et al., 1999). In this study, we created a mouse strain with profound HL (the equivalent of Otof^–/–^) through a point mutation approach (frameshift mutation). The Otof^–/–^ mice were detected by ABR testing at the ages of P14, P56, and P168, respectively, and there were no visual ABR waveforms at any checking points. On the other hand, normal waveforms were seen in the control mice (Figure 1A). It is consistent with the hearing detection above; there are no positive immunostaining signals of otoferlin in the cochlear hair cells of Otof^–/–^ mice (Figure 1C), compared with normal expression in control mice (Figure 1B), indicating that we obtained a suitable mice model for eliminating peripheral sound signal input before the onset of hearing. It has been reported that otoferlin could have an expression in the brain (Wu et al., 2015); however, we performed otoferlin immunostaining on hippocampal slices of WT mice, and we did not identify clear positive signals of otoferlin expression. In addition, it was reported that in an *in vitro* experiment, a deficiency of otoferlin in hippocampal neurons does not impair its presynaptic exocytosis function (Reisinger et al., 2011). Thus, our study excluded the possible effect that otof knockout in hippocampal neurons could disrupt social memory.

Social communication in rodents primarily depends on auditory, olfactory, and tactile feedback. Deprivation of sound information reduces the communication capacity and attenuates social memory. Somatosensory experiences can reorganize the cortical neural plasticity; similarly, cortical plasticity could be disrupted by long-term sensory deprivation (Merabet and Pascual-Leone, 2010). Our previous study showed that aberrant neural activity in the central auditory pathway is often accompanied by abnormal neural activity in the limbic system, especially in the hippocampus and amygdala. Therefore, it could have a functional connectivity between the central auditory pathway and the limbic system (Qu et al., 2019). It has been proposed that neuropsychiatric disorders may have social memory deficits, such as schizophrenia and autism (Meltzer et al., 1996). Thus, HL can induce alterations in social circuit neuroplasticity.

The hippocampal dorsal CA2 (dCA2) has been proved as an essential subfield in social memory (Hitti and Siegelbaum, 2014). The CA2 is highly expressed with the vasopressin 1b (Avpr1b) receptor (Young et al., 2006), and the knockout of Avpr1b results in social memory impairment (Wersinger et al., 2002; DeVito et al., 2009), whereas targeted activation of CA2 Avpr 1b during the acquisition period could enhance social memory (Smith et al., 2016), demonstrating their association. Dorsal CA2 projects to ventral CA1 (vCA1) (Meira et al., 2018), which then projects to the nucleus accumbens (NAc) also contributing to social memory storage, and vCA1 neurons greatly respond to familiar conspecifics than a novel mouse in the social memory test (Okuyama et al., 2016). Social isolation was shown to decrease the number of parvalbumin interneurons in the vCA1 and then disrupt social memory retrieval in mice (Deng et al., 2019). vCA1 projection to the medial prefrontal cortex (mPFC) and the NAc also play a critical role in social memory recall (Phillips et al., 2019; Xing et al., 2021). However, little is known about the underlying mechanism of how hearing deprivation disrupts neural circuits engaged in social memory. In this study, we completely eliminated hearing input from the cochlea to the brain and demonstrated that hearing input is truly necessary for the development of social memory. We hypothesize that hearing deprivation may reorganize functional connectivity between the auditory pathway and social memory circuit, resulting in negative effects on associated brain regions, such as the hippocampus (dCA2, vCA1), mPFC, NAc, and amygdala (Kwon et al., 2021). Also, HL can partially induce chronic inactivation of social memory-related networks, which in turn leads to discrimination failure between familiar and novel conspecifics in deaf mice. However, the underlying mechanism requires further investigation.

In our study, the deaf mice showed normal hippocampal-dependent spatial memory and novel object recognition, indicating that HL may have no significant effects on these targeted functions. However, some studies have shown that hearing impairment in adult rodents, induced by noise exposure, can impair hippocampal-dependent spatial learning and memory through decreased neurogenesis (Kraus et al., 2010; Liu et al., 2016, 2018; Zhuang et al., 2020). A possible explanation is that, in our study, auditory input has been entirely eliminated before the onset of hearing through genetic manipulation, and the reorganization of neural circuits occurred in its critical period in early development and thus exhibited normal general hippocampus functions by employing more cognitive resources during development. By contrast, noise trauma only reduced or destroyed hearing function in adult mice, which could have greatly disrupted the development of hippocampal-dependent spatial learning and memory in mice. Moreover, before the noise exposure, these mice still had the capacity to sense and deliver hearing input from the cochlea to the brain. Nonetheless, our results are consistent with those of a previous study indicating that HL in middle age is more likely to induce dementia (Griffiths et al., 2020). Our study has a significant limitation of not identifying the neural mechanism that is responsible for the association between HL and social memory impairment, and further studies addressing this issue need to be conducted.

## Conclusion

Our study found through a three-chamber social interaction assay that deprivation of peripheral auditory input before the onset of hearing can lead to social memory impairment in mice. Furthermore, we verified the relationship between hearing stimuli and hippocampal-associated cognitive function. Taken together, our findings demonstrated that hearing input is required for social memory since the initial development of hearing function in mice. In this study, we have established a mouse model with complete elimination of hearing input in the early stage of development. Prospectively, different types of mouse model with a distinct degree of HL might be applied to investigate the mechanisms underlying the associations of hearing input and memory.

## Data Availability Statement

The original contributions presented in the study are included in the article/supplementary material, further inquiries can be directed to the corresponding authors.

## Ethics Statement

The animal study was reviewed and approved by the Animal Ethics Committee of the Capital Medical University.

## Author Contributions

YL and KL contributed to the design of the study, analyzed and interpreted the result, and wrote the manuscript. SG contributed to the design of the study. YL and RG performed the experiments and analyzed the data. JL contributed to the data collection. All authors reviewed and approved the final version of the manuscript.

## Conflict of Interest

The authors declare that the research was conducted in the absence of any commercial or financial relationships that could be construed as a potential conflict of interest.

## Publisher’s Note

All claims expressed in this article are solely those of the authors and do not necessarily represent those of their affiliated organizations, or those of the publisher, the editors and the reviewers. Any product that may be evaluated in this article, or claim that may be made by its manufacturer, is not guaranteed or endorsed by the publisher.
